# Comparison of target labeling methods for use with Affymetrix GeneChips

**DOI:** 10.1186/1472-6750-7-24

**Published:** 2007-05-18

**Authors:** William Lonergan, Toni Whistler, Suzanne D Vernon

**Affiliations:** 1 Chronic Viral Diseases Branch, Division of Viral and Rickettsial Diseases, Centers for Disease Control and Prevention, 1600 Clifton Rd, MS-G41, Atlanta, GA, 30329, USA

## Abstract

**Background:**

Several different commercial one-cycle labeling kits are available for preparation of the target for use with the Affymetrix GeneChip platform. However, there have been no evaluations of these different kits to determine if comparable results were generated. We report on the cRNA target synthesis, labeling efficiency and hybridization results using the One-Cycle Target Labeling Assay™ (Affymetrix), the BioArray RNA Amplification and Labeling System™ (Enzo Life Sciences), and the Superscript RNA Amplification System (Invitrogen Life Technologies).

**Results:**

The only notable difference between kits was in the yield of cRNA target synthesized during in vitro transcription, where the BioArray assay had to be repeated several times in order to have sufficient target. However, each kit resulted in comparable signal and detection calls when hybridized to the Affymetrix GeneChip.

**Conclusion:**

These 3 one-cycle labeling kits produce comparable hybridization results. This provides users with several kit options and flexibility when using the Affymetrix system.

## Background

There are several commercially available one-cycle labeling kits that generate targets for use with Affymetrix GeneChip technology. Commercial labeling kits are valuable because they eliminate the need for individual laboratories to optimize methods, saving both time and resources. They also allow better cross-comparison of results generated from different laboratories. However, this assumes that all approaches produce comparable labeled targets. Given the widespread use of several labeling kits with the Affymetrix technology, we evaluated cRNA target synthesis, labeling and hybridization results using 3 different one-cycle linear amplification labeling kits. These were the One-Cycle Target Labeling Assay from Affymetrix (One-Cycle), the BioArray™ RNA Amplification and Labeling System from Enzo Life Sciences (BioArray), and the Superscript™ RNA Amplification System from Invitrogen Life Technologies (Superscript). Several steps for synthesizing labeled target are identical for each kit. For example, each kit uses reverse transcriptase with an anchored oligo(dT) primer containing a T7 promoter to synthesize first-strand cDNA. Then, following second strand synthesis, the cDNA templates are amplified via the Eberwine isothermal protocol [1]. Some of the differences include the biotinylated nucleotides in the *in vitro *transcription (IVT) reaction. The BioArray kit uses two biotinylated nucleotides; One-Cycle, a biotinylated pseudo-nucleotide and the Superscript kit a single biotinylated nucleotide. Differences are also noted in the second strand cDNA synthesis. The One-Cycle and Superscript kits use nick translation, whereas the BioArray kit uses a homopolymeric tailing and an extension reaction. In our study peripheral blood mononuclear cell (PBMC) RNA from 2 donors was used in each of the three labeling kits. Several parameters of these one-cycle labeling kits were compared to determine factors that could affect gene expression results.

## Results

Each kit was used to label 2.5 μg total RNA extracted from PBMC from two donors (A and B). The labeling reactions were repeated for Donor A, resulting in 3 GeneChip arrays for each kit.

**Figure 1:**
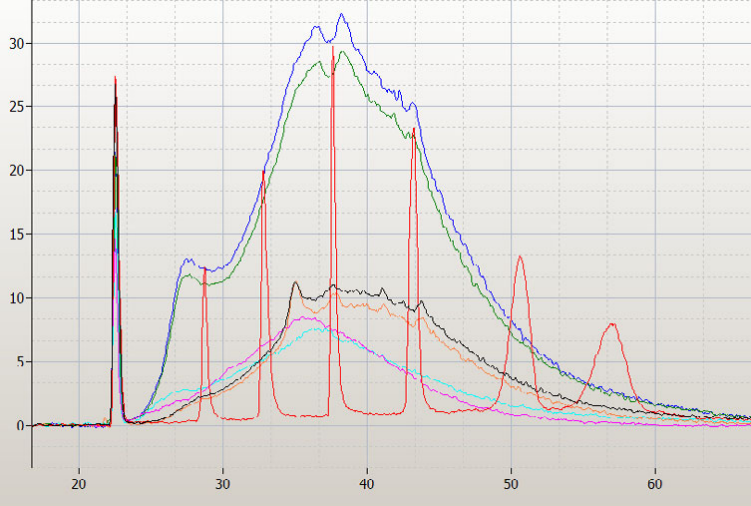
Overlaid electropherograms from the analysis of unfragmented biotinylated cRNA products from the IVT reactions of the 3 different labeling kits by the Agilent 2100 Bioanalyzer. The replicate reactions from donor A are shown for each kit: One-Cycle data represented as blue and green line; BioArray as black and orange and Superscript by the pink and turquoise lines. 1 μl of the final volume (One-Cycle = 21 μl; BioArray = 60 μl; Superscript = 100 μl) of purified IVT reaction is loaded. The RNA ladder (peaks represented in red) contains a mixture of RNAs of known concentration and size (50 (lower marker) 200, 500, 1,000, 2,000, 4,000, and 6,000 bases from left to right).

The length of the synthesized cRNA targets was estimated by overlaying the Bioanalyzer profiles onto the standard size markers (Figure 1). Determinations were made at a y-axis (fluorescence) value of 2 (twice the background level). The sizes ranged from 100 to 5000 nucleotides for the One-Cycle kit; 200 to 4000 nucleotides for the Superscript kit, and 200 to 2000 nucleotides for BioArray kit (Figure 1). On correction for different loading volumes similar results were obtained. The cRNA yields were quite different. The Superscript kit synthesized the highest labeled amounts and the BioArray kit the lowest (Table 1). Importantly, the BioArray methodology needed to be repeated several times in order to obtain the required yield from the IVT reaction. Approximately 50% of these reactions failed to give an appropriate yield (data not shown) and we do not believe this was user error.

**Figure 2:**
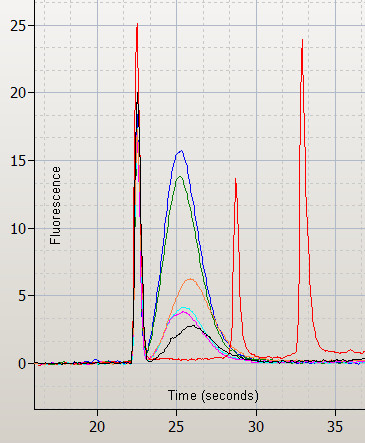
Overlaid Bioanalyzer electropherograms for fragmented labeled cRNA targets showing the size distribution of fragmented target. One-Cycle replicates (blue and green); Superscript replicates (orange and black); BioArray replicates (pink and turquoise). RNA ladder in red showing the lower alignment marker and the 200 and 500 base markers.

The fragmentation of the cRNA target to 35 to 200 bases is important for hybridization efficiency and assay sensitivity [2]. The fragmentation protocol recommended by Affymetrix was used in all cases. Approximately 160 ng of the fragmented target (estimated from concentration of starting material) was assessed using the Agilent 2100 Bioanalyzer to determine population size relative to known markers (Figure 2). The One-Cycle and Superscript kits produced cRNA fragments with an average size of around 100 bases. The BioArray kit produced a slightly larger average size fragment of 125 bases. Even though the starting concentration for each fragmentation reaction was the same, the One-Cycle kit routinely gave almost double the yield of fragmented cRNA than either the BioArray or Superscript kits (Table 1).

### Hybridization performance with standard array quality metrics

To verify the efficiency of the hybridization step we examined the results of the hybridization spike-in controls provided for the Affymetrix GeneChip (QC1). These controls are a mixture of biotin-labeled cRNA transcripts of *bioB*, *bioC*, *bioD*, and *cre*, prepared in staggered concentrations (1.5 pM, 5 pM, 25 pM, and 100 pM respectively). These are added into the hybridization cocktail, independent of RNA sample preparation, and are used to evaluate hybridization efficiency. Not surprisingly the hybridization performed equally well for the 3 kits as all control targets were called present in all arrays and there was a linear increase in signal with increasing concentrations of each control cRNA transcript.

The background, RawQ and scaling factor quality metrics were examined to determine if differences in labeling affected the hybridization results. The average background values, derived from the intensity values of the lowest 2% of cells on the chip, were within the recommended 20–100 range for all 3 approaches. The Superscript kit consistently gave higher readings with larger variation (Table 1). The RawQ (or noise) value, derived from the standard deviation of the background intensity measurement was below 4 for each kit, as recommended by Affymetrix. The scaling factor is inversely related to the overall brightness of the chip and provides a measure of the overall expression level of the array and was again, comparable for all 3 labeling approaches (Table 1).

**Figure 3:**
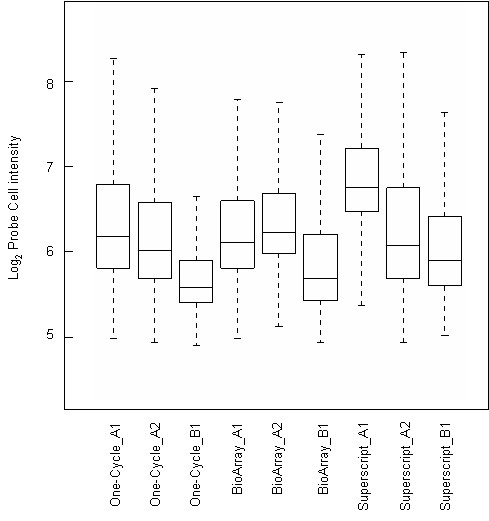
Box plots of logged probe level signal intensities for each of the GeneChip arrays included in this study.

**Figure 4:**
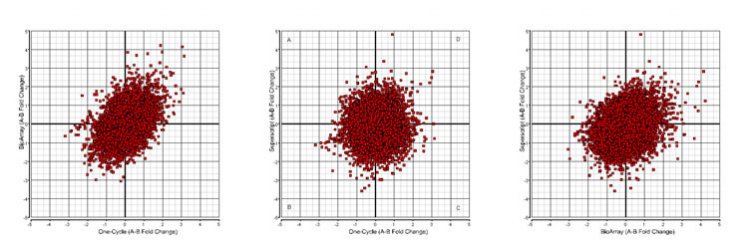
Correlation of fold-change calculations across different labeling kits. Fold-changes were calculated for probesets called present (11881 probesets) in all samples between Donor A and Donor B for each labeling kit. Quadrants B and D represent data points that are concordant with respect to direction of fold change. Quadrants A and C represent discordant fold change results. Left: One-cycle vs. BioArray. Center: One-Cycle vs. Superscript. Right: BioArray vs. Superscript.

The percent present/absent calls were used to globally assess the sensitivity of each labeling kit. The majority of genes were consistently classified in the same category for each labeling pair (88.6% for One-Cycle, 88.2% for BioArray and 84.6% for Superscript). There was 74.2% call agreement across the 3 methods (40,548 out of 54,675 probesets on the U133Plus2.0 GeneChip). Interestingly, even though One-Cycle kit had the higher percent present calls of all the kits, all had similar average signal intensities (Table 1). Signal intensities for the probe sets are comparable, but the Superscript kit has the highest variation (Figure 3). A comparison of coefficients of variation on normalized signal intensities shows an average 11% CV for the One-Cycle kit compared to 12.1% and 12.3% for BioArray and Superscript kits respectively (Table 1). When we examine the correlation of differential fold change calculations (between Donor A and Donor B) using all 54675 probesets across the labeling kits we find a 60.2% agreement between the One-Cycle and BioArray kits, (number of probesets present in quadrants B and D (Figure 4) compared to the total number of probesets). A 53.9% concordance is seen between the One-Cycle and Superscript kits and 63% between the BioArray and Superscript kits. This same analysis applied to data filtered for present calls across all 6 arrays (11881 probesets, Figure 4) showed an increase in concordance between the One-Cycle and BioArray kits (65.7%) with a linear regression fit of R^2 ^= 0.43. For the two other combinations the percentage decreased to 51% for the One-Cycle/Superscript combination (R^2 ^= 0.02) and remained the same for the BioArray/Superscript comparison (R^2 ^= 0.3).

**Figure 5:**
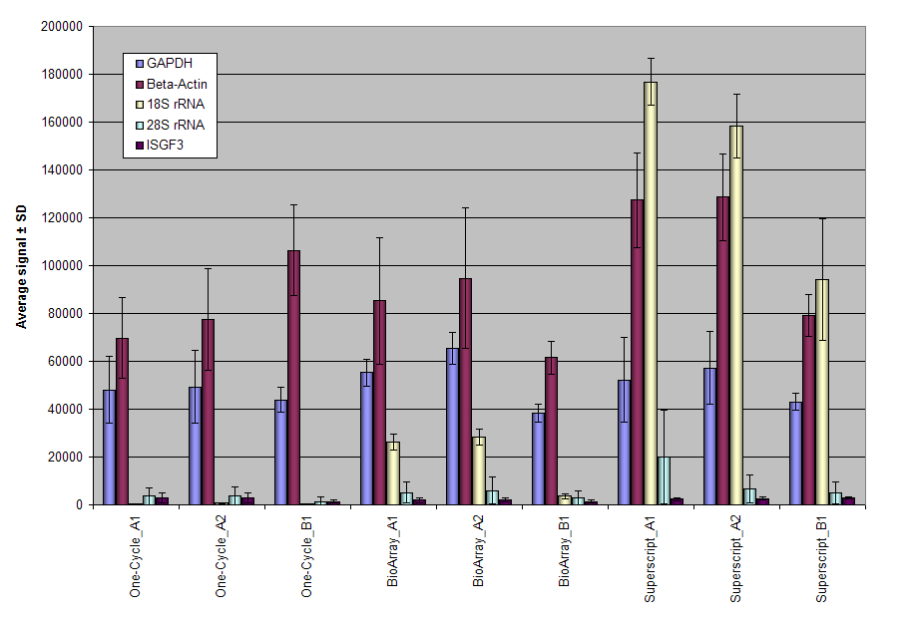
Bar chart showing averaged signal intensities for all internal control probesets (QC2) with corresponding standard deviations.

Table 2 shows the averaged 3'/5' ratios for the internal control genes, QC2, and gives the total distance covered by the probesets for each. GAPDH and β-actin have the shortest transcript lengths and Affymetrix suggests that ratios < 3 are acceptable, as data quality is not significantly affected when the ratios fall within these bounds [3]. All 3 methodologies met these criteria. There are probesets directed to the 28S and 18S ribosomal RNA (rRNA) genes, as these are not mRNAs they can be considered as negative controls. Figure 5 shows a bar chart of the averaged signal intensities for all the internal control gene probesets, QC2. Signal intensities for the rRNA probesets from One-Cycle are lower than either BioArray or Superscript. However, all probesets (3', middle and 5') were called as present, except the 3' 28S rRNA which was absent on all 9 chips. ISGF-3 (Interferon-simulated gene factor 3) mRNA is the longest transcript for the internal control genes and has ratios = 3 for the One-Cycle and BioArray methodologies, and 1.5 for the Superscript kit.

### Reproducibility of gene expression signal intensities

**Figure 6:**
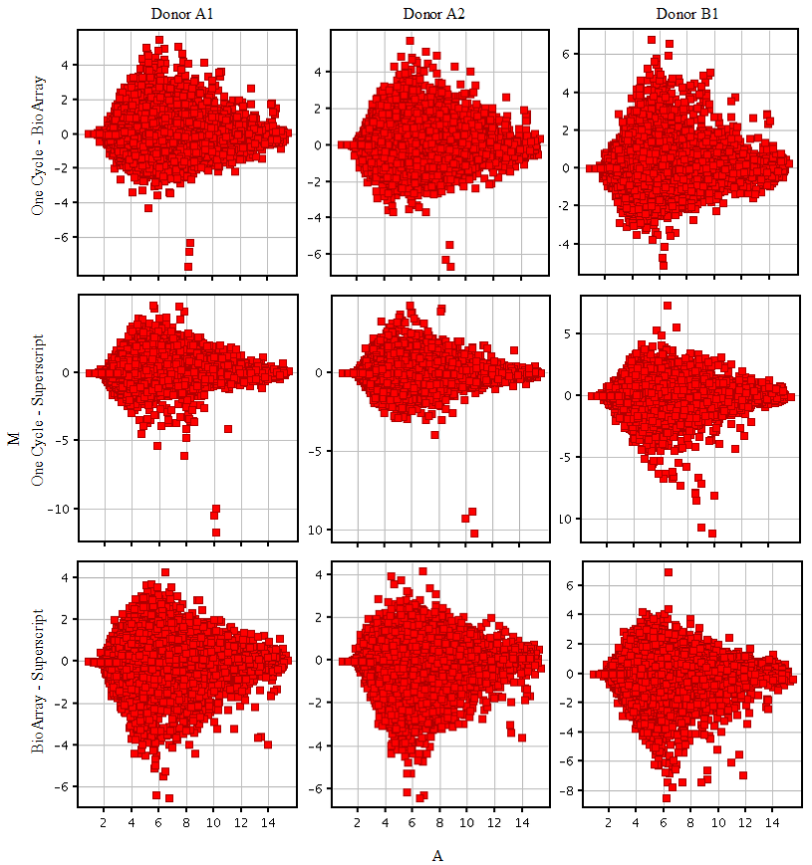
MA plots comparing the magnitude of change (log(signal array1) - log(signal array2)) on the y axis against the average log signal intensity (x axis). The green threshold lines show ± 2-fold changes. The color coding of the plot indicates the density of probesrepresented by that data point. The kits compared in each plot are given on the right hand side and the donor sample labeled by the kit is indicated at the top of each plot.

A compilation of the MA plots comparing the magnitudes of change (log fold change) to the mean log expression level for the same sample labeled by each kit is given in Figure 6.

Intra- and inter-assay comparisons were determined using the overall signal correlation. Pair-wise Pearson correlation coefficients of the raw signal intensities were calculated using all transcripts. The intra-method reproducibility for replicate samples had correlation coefficients >0.99 for One-Cycle and BioArray kits and 0.98 for the Superscript kit. The One-Cycle and BioArray methods had the highest inter-method correlation coefficient (averaged r = 0.972) whereas the Superscript kit was less correlated (average r = 0.968 against One-Cycle and BioArray). The inter-assay correlation range was 0.943 – 0.986. When we examine the non-parametric Spearman Rank correlation coefficients generated for the normalized probesets within the replicate samples One-Cycle has the highest correlation (r = 0.936), followed by BioArray (r = 0.911) and then Superscript (r = 0.906). As the Spearman correlation ranks the probesets by their intensity values, this reflects the reproducibility of the labeling technologies.

### Linearity and sensitivity of kits as quantified using spike-in bacterial poly-A control targets

**Figure 7:**
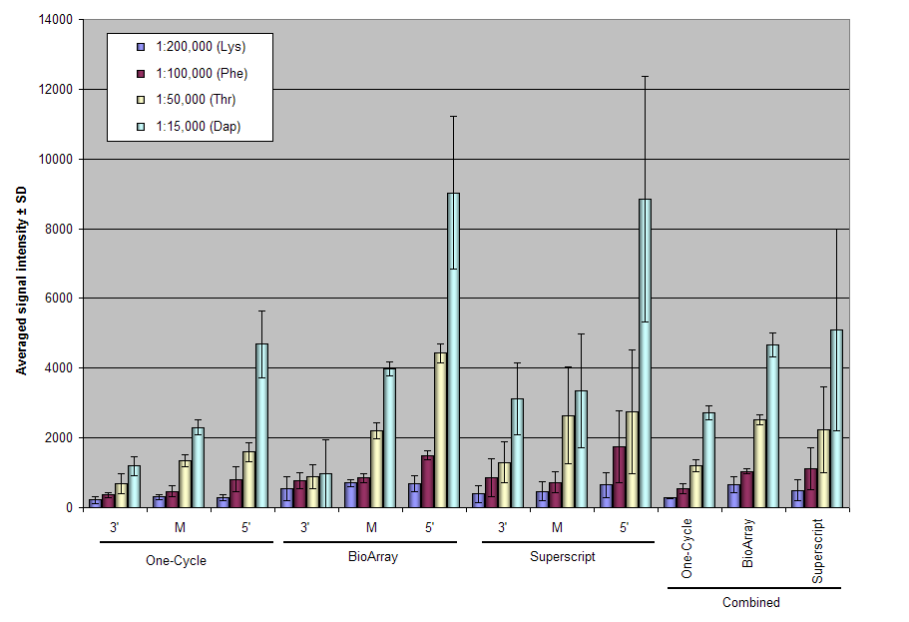
Evaluation of transcripts spiked into the total RNA sample (poly-A controls, QC3). Averaged signal intensity of each of the 3 GeneChips showing the standard deviation.

The ability of any amplification and labeling method to accurately detect differences in expression levels is highly dependent on linearity, dynamic range of amplification and sensitivity. We evaluated these parameters by analyzing the spike-in poly-A control transcripts (QC3) added to each total RNA sample at staggered concentrations. They act as a convenient way to monitor the entire labeling process, independent of the quality of the starting material. All 4 spike-in controls were called Present in all instances, indicating similar sensitivities. The averaged signal intensities for the poly-A controls were plotted (Figure 7) for each of the probesets, (3', middle and 5'). Each followed the same order of increasing poly-A control RNA concentration as expected.

## Discussion

Total RNA is ideal for gene expression profiling from clinical specimens, small amounts can be used because the methodology preserves the relative abundance of the different mRNAs in the original sample [4]. In evaluating a new protocol or comparing existing protocols, measures such as the yield of cRNA or the fraction of probe sets detected can be useful, but the key measure is the extent to which differences (or in this analysis similarities) in gene expression can be detected. Several studies have compared linear amplification protocols, similar to those used here, with PCR-based techniques [5,6], or one-cycle labeling kits to those that use two or three cycles for sample of 100 ng or less total RNA [7]. Ma *et al*. (2006) [8] compared IVT labeling reactions from purified double-stranded cDNA using the GE Healthcare CodeLink Expression System and the 3'-amplification reagents from Affymetrix, showing similar results to those obtained here. However, a literature search revealed no publications comparing one-cycle labeling kits directly. To examine the differences introduced by different labeling kits, we compared 3 one-cycle labeling kits commonly used with the Affymetrix GeneChip platform. Each labeling method showed a high degree of intra-method correlations in replicate experiments, but lower inter-method correlation. Exploring the fold changes between Donor A and Donor B across kits (Figure 4) and the MA plots (Figure 5) showed the One-Cycle and BioArray kits, gave the best correlation. The poorest concordance was evidenced between the two Eberwine-based protocols, One-Cycle and Superscript.

The use of the oligo(dT) primer in the first strand synthesis in all the kits is to selectively amplify polyadenylated transcripts. Therefore the ribosomal RNA probesets (to 18S and 28S rRNA) act as a negative control. As expected for these probesets, the One-Cycle kit gave a signal just above background, however, for both the BioArray and Superscript kits a strong positive signal was detected for the 18S rRNA probesets (Figure 7). This is of concern, as any signal detected comes from either cross-hybridization or non-specific priming. We believe these results can be explained by the rapid cooling of the primer annealing reaction in the One-Cycle methodology which reduces some of the non-specific binding that may arise with the slower cooling used in the Superscript or BioArray protocols.

β-actin and GAPDH internal control probesets are commonly used to assess RNA sample and assay quality [3,9,10]. The other probesets classified as internal controls are rarely referred to in the literature, probably because the two rRNA probesets (discussed above), are known to have very high coefficients of variation [11] and the ISGF3 probesets cover 2639 nucleotides of the mRNA, a particularly long transcript for Affymetrix GeneChips.

Other possible causes for the differences in results between the labeling kits are the use of different reverse transcriptase enzymes, T7oligo dT primers (which could differ in promoter sequence and length of poly-T tail) and concentration of nucleotides. BioArray uses a very different strategy to generate the second strand in the cDNA synthesis – a homopolymeric tail to the cDNA, followed by an extension reaction. The other kits use a nick-translation reaction. The IVT reaction for amplification and labeling of the target is similar in most aspects, except the BioArray method uses two nucleotide labels (biotin-CTP and biotin-UTP), the Superscript kit a single biotin-UTP labeling and the One-Cycle, a ψ-UTP biotinylated nucleotide (Table 3). Samples labeled by the BioArray method had higher, unnormalized fluorescence intensity values than the other methods (Table 1), possibly due to incorporation of the two labeled nucleotides. The higher number of present calls on the One-Cycle kit array indicates a higher labeling efficiency, but this did not appear to be because of greater label incorporation, as signal intensities were no higher in the One-Cycle kit arrays compared to the other two.

The methodological differences elucidated above, will each make a contribution towards the differences in cRNA profiles that were noted when an aliquot of each was run on the Bioanalyzer (Figure 1). These differences were not a result of loading different quantities of cRNA on the Bioanalyzer chips, as similar results were obtained when equivalent amounts of cRNA were run. The differences included length of product, yield and reproducibility of the methodology. A cRNA profile with a greater proportion of fragments above 500 nt is usually considered as a good preparation, therefore despite the differences, each of the kits provides target cRNA that passes the suggested parameters established by Affymetrix and our results support this. Once the biotinylated target cRNA has been synthesized, it is then fragmented by metal-induced hydrolysis. Considering that a set amount of cRNA goes into each reaction (20 μg) and the same protocol was used for all the fragmentations, we were surprised to note the differences in the fragmentation profiles for each kit. The BioArray and Superscript protocols give far smaller yields when compared to the One-Cycle kit cRNA. The 3 kits each use spin-cartridges with differing reagents to clean-up the cRNA, and the product is eluted in RNAse-free water in each case. The BioArray and Superscript kits require that the target be concentrated in a vacuum concentrator for fragmentation. We cannot be sure what the carry-over (i.e. ion concentration) is from these steps, and it is possible that this could affect the fragmentation reaction, possibly causing complete hydrolysis and loss of product.

Probably the largest draw back that we experienced with any of the kits was the number of times that the BioArray kit failed to produce sufficient product for fragmentation (50% of reactions). This is probably due to losses incurred from the two additional purification steps (after the reverse transcription and again after the homopolymeric tailing) in this methodology. This is not an acceptable situation when processing limited clinical samples. Enzo Life Sciences has recently released a new labeling kit based on the Eberwine protocol, which they currently recommend for use with Affymetrix GeneChips. Other considerations we used in evaluating the kits were ease of use, measured by the number of steps involved in the protocol and cost of each labeling reaction (Table 2).

## Conclusion

The results of this study show that different one cycle labeling kits from different manufacturers generate products that vary in size distribution and yield. The One-Cycle Target Labeling Assay from Affymetrix and the Superscript™ RNA Amplification System from Invitrogen Life Technologies each use the nick translation methodology for generation of second-strand cDNA and were the most similar in terms of product generated. Whereas the BioArray™ RNA Amplification and Labeling System from Enzo Life Sciences uses an extension reaction after homopolymeric tails are added, and this kit produces a quite different product profile. The results of hybridizing each of the labeling kit products to Affymetrix GeneChip arrays showed much less variation in terms of gene expression results than expected from the product profiles. The One-Cycle and BioArray kits produced the most concordant data. Since the results generated from the different labeling kits are fairly comparable, factors such as kit cost, time and difficulty should be considered when selecting a one-cycle labeling approach for use with Affymetrix GeneChips. These results emphasize that data generated from different labeling methodologies cannot be directly compared.

## Methods

### Sample processing

Peripheral blood mononuclear cells were used as the source of total RNA for these experiments. Blood was drawn from two donors (Donor A and B) into 8 ml Vacutainer Cell Preparation Tubes with sodium citrate (BD, NJ), and immediately processed according to manufacturer's instructions. Total RNA was isolated using TRIzol™ Reagent (Invitrogen, CA). Integrity and concentration of the RNA were evaluated using the Agilent Bioanalyzer 2100 capillary electrophoresis RNA 6000 Nano Kit (Agilent Technologies, CA). RNA with a 28S:18S RNA ratio greater than 1.8 was used in this study.

### Target preparation

Three commercially available target labeling kits compatible with the Affymetrix GeneChip platform were assessed in this study. These were: One-Cycle Target Labeling Assay from Affymetrix ((One-Cycle), Santa Clara, CA) [2], which includes the sample clean-up modules; BioArray™ RNA Amplification and Labeling System (with biotin-labeled ribonucleotide mix) from Enzo Life Sciences ((BioArray), Farmingdale, NY) [12] used along with the RNeasy Mini and MinElute Kits (Qiagen, Valencia, CA) for purification of reaction products; and Superscript™ RNA Amplification System from Invitrogen Life Technologies ((Superscript), Carlsbad, CA) [13] incorporating the modifications for GeneChip applications as suggested in the manual using biotin-11-UTP purchased from Perkin- Elmer (Boston, MA).

Each labeling reaction used 2.5 μg total RNA and was completed by the same researcher to minimize user-associated variability. Three reactions were performed per kit: two using Donor A to assess technical variation, and a third using RNA from Donor B. The Eukaryotic Poly-A RNA Control Kit designed by Affymetrix to provide exogenous positive controls to monitor the entire target labeling process (QC3) were used with each of the 3 kits. Poly-A RNA controls were processed as directed for 5 μg total RNA immediately before performing the experiment. This doubled the final concentration of the spike-in controls to the total RNA. The methods compared, are summarized in Table 3. The cRNA amplification products from the IVT reaction (1 ul from final purification volume) were examined on the Agilent Bioanalyzer to obtain the size distribution and yield.

### Fragmentation and hybridization of labeled target

Twenty μg of purified cRNA was incubated in Affymetrix fragmentation buffer at 94°C for 35 minutes and 150 ng of fragmented target was assessed using the RNA 6000 Nano Kit, Agilent 2100 Bioanalyzer. Hybridization buffer, the Eukaryotic Hybridization Controls (used to confirm the sensitivity of hybridization, QC1), and the OligoB2 controls (positive controls used to orient and grid the array), were added to the fragmented cRNAs. Labeled targets were hybridized on Affymetrix Human U133 plus 2.0 chips at 45°C for 16 hours as described in the Affymetrix Users manual [2]. Washing and staining of arrays were performed using the GeneChip Fluidics Station with the EukGE-WS2v5_450 protocol. Chips were scanned using the Affymetrix GeneChip Scanner 3000.

### Analysis of gene expression data

Image acquisition, quantification and data analysis were performed using the Affymetrix GeneChip Operating Software (GCOS) v1.4.0.036. Each sample was scaled to a target intensity of 500 for all probe sets. This option scales the trimmed mean intensity to the specified value. The expression report generated using the GCOS software was used to examine raw signal values and detection calls (present (P), absent (A), marginal (M). Data was normalized using the MAS5 algorithm.

All gene expression data has been deposited in the ArrayExpress repository [14] under the accession number E-MEXP-884, and is available to the public.

## Abbreviations

cDNA complementary deoxyribonucleic acid

IVT *in vitro *transcription

cRNA complementary ribonucleic acid

ng nanogram

mRNA messenger ribonucleic acid

rRNA ribosomal ribonucleic acid

CA California

NY New York

UTP uridine triphosphate

aRNA amplified ribonucleic acid

GCOS GeneChip Operating Software

PBMC peripheral blood mononuclear cells

## Authors' contributions

WL was responsible for the laboratory component of the study and acquisition of data. TW was involved in the analysis and preparation of the manuscript. SDV was responsible for critical assessment and revision of the manuscript.

All authors have read and approve this manuscript.

## Figures and Tables

**Table 1:** Comparison of different measures for each of the 3 kits assessed in this study.

Stage	Parameters	One Cycle	Rank	BioArray	Rank	Superscript	Rank
Labeling Protocol	cRNA yield (ug)*	54.6 ± 0.6	2	43.1 ± 2.4	3	86.3 ± 10.9	1
	Reproducibility		1		2		3
	Peak range cDNA product sizes (bases)	100 – 2000	1	800	3	750–2000	2
	Average fragment length (bases)	100	1	125	1	100	1
	Fragmentation cRNA yield (ug)*	9.2 ± 4.3	1	4.6 ± 1.2	2	4.5 ± 1.3	2
	Total time labeling reaction	22.5	1	23.5	3	22.75	2
	Number of steps to labeling reaction	7	1	11	2	6	1
	Cost per labeling reaction	$115.00	3	$106.00	2	$95.00	1
Signal measures	% of Present*	38.6 ± 0.7	1	36.8 ± 5.4	2	34.7 ± 6.4	3
	Scaling Factor*	16.6 ± 7.7	3	13.7 ± 1.9	1	14.0 ± 1.4	2
	Median array raw intensity*	143.1 ± 8.7	2	131.6 ± 33.6	3	168.6 ± 47.0	1
	Mean array raw intensity*	1042.6 ± 34.3	3	1160.7 ± 127.5	1	1104.6 ± 73.5	2
	Background*	37.4 ± 3.8	1	41.8 ± 8.6	2	49.9 ± 21.9	3
	Raw Q (Noise)*	1.23 ± 0.2	1	1.4 ± 0.5	2	1.7 ± 0.8	3
	Number Present (P)	21099 ± 389	1	20119 ± 2962	2	18953 ± 3477	3
	Number Absent (A)	32744 ± 360		3372 ± 2933		34846 ± 3461	
	Number Marginal (M)	832 ± 81	1	834 ± 13	2	877 ± 16	3
	Average Signal (P)	2550.4 ± 125.4	3	3054.3 ± 660.8	2	3074.7 ± 451.1	1
	Average Signal (A)	90.5 ± 12.5		88.8 ± 35.3		143.9 ± 42.7	
	Average Signal (M)	295.5 ± 36.0		279.5 ± 105.9		430.3 ± 121.6	
	Average CV on GC-RMA normalized signal (All 3)	15.7%		18.0%		16.0%	
	Average CV on GC-RMA normalized signal (Replicates only)	11.0%	1	12.1%	2	12.3%	3
Internal Control genes	β-actin/GAPDH ratios		1		1		1
	rRNA signal		1		3		2
Total score			30		41		40

Several criteria were evaluated for each stage in the generation of labeled target and each afforded a score relative to performance 1 = good, 3 = worst.

**Table 2:** 3'/5' ratios of Housekeeping genes (internal control genes, QC2) given as average ± standard deviation.

Internal Control Genes			3'/5' Signal Ratios (Average ± SD)		
Probe ID	Gene	Length Transcript (bases)	Affy	Enzo	Invitro

AFFX-HUMGAPDH/M33197	GAPDH	1169	1.5 ± 0.2	1.1 ± 0.2	1.5 ± 0.3
AFFX-HSAC07/X00351	beta-Actin	1688	1.6 ± 0.2	1.5 ± 0.2	1.2 ± 0.1
AFFX-HUMRGE/M10098	18S rRNA	1795	0.9 ± 0.2	1.0 ± 0.2	0.8 ± 0.1
AFFX-M27830	28S rRNA	1816	0.03 ± 0.01*	0.02*	0*
AFFX-HUMISGF3A/M97935	ISGF3	2639	4.2 ± 1.8	3.0 ± 0.3	1.5 ± 0.1

* 3' signal called absent

**Table 3:** Comparison of labeling methodologies for kits compared in this study.

**Stage**	**Steps**	**One-Cycle**	**Time (hours)**	**BioArray**	**Time (hours)**	**Superscript**	**Time (hours)**
	Optimized Total RNA (μg)	1 to 15		1 to 10		1 to 5	
First Strand Synthesis	Total RNA used	2.5 μg	1.5	2.5 μg	1.25	2.5 μg	2.5
	Primer	T7-oligo(dT)		T7-oligo(dT)		T7-oligo(dT)	
	Primer annealing/RNA denaturation	70°C × 10' and 4°C × 2'		70°C × 10'		70°C × 10'	
	Reverse transcriptase	Superscript II		Proprietary		Superscript III	
	Final reaction volume	20 μl		20 μl		20 μl	
	Incubation conditions	42°C × 1 h		42°C × 1 h		46°C × 2 h	
	Inactivate RT	-		-		70°C × 10'	
	RNA elimination	-		Base hydrolysis/Neutralization	0.5	-	
	Purification	-		Spin cartridge 1	0.5	-	
Tailing and Termination	Methodology	-		Homopolymeric tail added	1	-	
	Purification	-		Spin cartridge 1	0.5	-	
Second Strand cDNA synthesis	Methodology	Nick translation reaction	2.5	Extension reaction	1.25	Nick translation reaction	2
	Volume used from previous reaction	All		All		All	
	Final reaction volume	150 μl		30 μl		150 μl in 2 tubes	
	Incubation conditions	16°C × 2 h		42°C × 1 h		16°C × 2 h	
	T4 DNA polymerase (sticky ends)	16°C × 5'		-		-	
	Stop reaction	EDTA addition		-		-	
	Purification	Spin cartridge	0.5	Spin cartridge 1	0.5	Spin cartridge	0.25
In vitro transcription amplification	Volume used from previous reaction	All	16.25	All	16.25	All	16.5
	Biotinylated nucleotide	Pseudouridine biotinylated nucleotide		Biotin-11-UTP/Biotin-11-CTP		Biotin-11-UTP	
	Final volume IVT	42 μl		40 μl		40 μl	
	Incubation conditions	37°C × 16 h		37°C × 16 h		37°C × 16 h	
	DNAse I digestion	-		-		37°C × 30'	
	Purification	Spin cartridge	0.5	Spin cartridge 2	0.5	Spin cartridge	0.25
	Volume after purification	21 μl		60 μl		100 μl	
	Methodology	Affymetrix		Affymetrix		Affymetrix	
Fragmentation protocol	Amplified RNA used	20 μg	1.25	20 μg	1.25	20 μg	1.25
	Final volume Fragmentation	40 μl		40 μl		40 μl	
	Total Hours		22.5		23.5		22.75
	Number of steps	7		11		6	

All details are taken from manufacturers manuals supplied with each kit.
